# The Interim Report or the Whisky Commission

**Published:** 1908-07-25

**Authors:** 


### The Interim Report of the Whisky Commission.

It will be familiar to all of our readers that prior to 1891 there was considerable commotion in the whisky trade owing to the largely increasing sale of so-called silent spirit, or whisky produced by the distillation, in a patent or Coffey still, of a fermented wort derived from malt and cereal grain. This spirit was at that time improperly termed "silent" by the manufacturers of strongly-flavoured whiskies, which were in most, but by no means all, cases derived entirely from malt, and were all distilled in an old-fashioned or pot still. The origin and growth in sale of patent still whisky was caused by the public demand for a mild as compared with a strong-flavoured whisky. The mild whisky was used to some extent by itself, but to a greater extent for blending or diluting the strong, heavy pot-still whiskies. This new product so upset the whisky trade that in 1891 a Select Committee was appointed, before which a certain faction of the trade argued that the spirit produced in a patent still, either when sold by itself or in blends of certain quantities, is falsely described as whisky, and that the term whisky should be entirely confined to the product of the pot still, which, on account of it containing a larger amount of impurities, has to be matured for a certain length of time before it was drinkable. The Select Committee of 1891 reported to the effect that they were unable to restrict the use of the name whisky to a spirit produced either from certain materials or by means of certain apparatus, provided the finished product had the taste of whisky and did not contain noxious ingredients.

The matter remained practically at rest, except that the sale of patent still whisky increased by leaps and bounds, until the summer of 1905, when the Borough of Islington proceeded under the Sale of Food and Drugs Act against certain publicans for selling as whisky a spirit consisting largely of patent-still spirit. The borough called medical evidence to the effect that while pot-still whisky is a valuable medicament, patent-still whisky is practically useless. In spite of strong evidence, both scientific and medical, on the other side, the Court upheld the contention of the Borough. This absurd verdict, against which appeal was made, was strongly commented upon in our columns (The Hospital, March 3, 1906). The appeal came on for hearing a few months afterwards, and after elaborate arguments on both sides had been heard, the Bench were unable to arrive at a decision. It thus fell out that the expenditure of thousands of pounds, much of it public money, left the question precisely where it was. An appeal was then made to the Local Government Board, and the appointment of the Royal Commission was promised. The interim report of this Commission was published last Friday, and is practically identical, as were its minutes of reference, with the report of the Select Committee of 1891. The conclusions at which the Commission arrives are that the term whisky can be correctly applied to a spirit produced by the distillation in either a pot or a patent still of a fermented wort derived either from malted barley alone or from malt and other cereal grain.

There can be no doubt in the mind of any reasonable person that this conclusion is both right from the point of view of the medical profession and the public health, and ethical from the point of view of the whisky trade. It is the view which we have always upheld in these columns throughout the controversy, after our inquiry into the subject by means of a Special Analytical Whisky Commission, whose report was published in The Hospital on April 7, 1906. From the medical standpoint the position of whisky, both before the Royal Commission and elsewhere, has been clearly defined by Dr. Tunnicliffe. Whisky is a preparation of alcohol: the harm that it does is due to the alcohol; the good that it does is due to the alcohol. The substances that it contains other than alcohol belong pharmaceutically either to the class of diluents (water) or adjuvants (flavouring agents). The best therapeutic results in different patients will be obtained by different whiskies, and practically by the whisky the individual patient prefers. The rich all-malt pot-still whisky, with a high content of higher alcohols and compound ethers, will be a suitable preparation of alcohol to give to a patient when grouse shooting in the Highlands, whereas the milder or blended spirit may suit him better when he is following his sedentary occupation in London.

